# S06-2: Moving Cancer Care: The importance of exercise during and after a cancer treatment

**DOI:** 10.1093/eurpub/ckae114.223

**Published:** 2024-09-26

**Authors:** Marie Crabbe, Dimitri Vrancken, Vicky Van Stappen, Ine Declerck

**Affiliations:** Artevelde University of Applied science, Belgium; Artevelde University of Applied science, Belgium; Artevelde University of Applied science, Belgium; Artevelde University of Applied science, Belgium

## Abstract

**Purpose:**

Literature is recognizing exercise in cancer as a promising therapeutic tool. Authors have shown that exercise could prevent health deterioration, reduce the risk of comorbidities, and improve overall quality of life. Despite this, few cancer patients participate in exercise programs. In this study, we identify barriers and solutions for cancer patients and professionals in promoting exercise. We investigate patient motivations, enhance professional roles (assess, motivate, refer) and identify optimal exercise programs.

**Methods:**

We used a human-centred design research, an iterative approach that focuses on understanding and addressing the needs of the field. For this, we first reviewed the international literature and specifically mapped the situation in Flanders (Belgium). We used qualitative (face-to-face interviews, focus groups, online survey) and quantitative (parameter registration) methods among cancer patients (39) and health/exercise professionals (36). In the second phase, our goal was to address the issue through collaborative feedback and partnership with the professional field. This resulted in the creation of practical tools, allowing professionals to assist cancer patients more effectively in their exercise routines.

**Results:**

Patients and professionals commonly face barriers related to exercise resistance (e.g. lack of motivation) and limited referral pathways.

Patients also express concerns about the location of exercise programs and susceptibility to external factors (e.g. weather conditions). While health and exercise professionals contend with challenges such as limited professional knowledge and organizational barriers, including an overloaded workload.

When developing exercise programs, it is important to incorporate proven design criteria from previous research. These include the use of behaviour change models, adherence to evidence-based exercise standards, and the provision of guidance and follow-up throughout the program.

**Conclusions:**

This research highlights the urgent need for a paradigm shift in cancer care, advocating not only more appropriate exercise programs, but also the integration of exercise coaching into the standard care pathway for all cancer patients.

The inspirational book and practical guidelines elaborated serves as valuable resources for health and exercise professionals and aims to promote physical activity during cancer treatment. This project aims to contribute to the future of care, envisioning sustainable physical rehabilitation beyond the traditional hospital setting

